# Reach&Grasp: a multimodal dataset of the whole upper-limb during simple and complex movements

**DOI:** 10.1038/s41597-025-04552-5

**Published:** 2025-02-07

**Authors:** Dario Di Domenico, Inna Forsiuk, Simon Müller-Cleve, Simone Tanzarella, Florencia Garro, Andrea Marinelli, Michele Canepa, Matteo Laffranchi, Michela Chiappalone, Chiara Bartolozzi, Lorenzo De Michieli, Nicolò Boccardo, Marianna Semprini

**Affiliations:** 1 Rehab Technologies Lab, Italian Institute of Technology, Via Morego, 30, 16163 Genova, GE Italy; 2 Department of Electronics and Telecommunications, Politecnico di Torino, Turin, 10124 Italy; 3 Event-Driven Perception, Italian Institute of Technology, Via San Quirico, 19, 16163 Genova, GE Italy; 4 Bioengineering Lab, University of Genova, DIBRIS, Genova, Italy; 5 Open University Affiliated Research Centre at Istituto Italiano di Tecnologia (ARC@IIT), Genova, Italy

**Keywords:** Biomedical engineering, Data publication and archiving, Data acquisition

## Abstract

Upper-limb movement characterization is crucial for many applications, from research on motor control, to the extraction of relevant features for driving active prostheses. While this is usually performed using electrophysiological and/or kinematic measurements only, the collection of tactile data during grasping movements could enrich the overall information about interaction with external environment. We provide a dataset collected from 10 healthy volunteers performing 16 tasks, including simple movements (i.e., hand opening/closing, wrist pronation/supination and flexion/extension, tridigital grasping, thumb abduction, cylindrical and spherical grasping) and more complex ones (i.e., reaching and grasping). The novelty consists in the inclusion of several types of recordings, namely electromyographic -both with bipolar and high-density configuration, kinematic-both with motion capture system and a sensorized glove, and tactile. The data is organized following the Brain Imaging Data Structure standard format and have been validated to ensure its reliability. It can be used to investigate upper-limb movements in physiological conditions, and to test sensor fusion approaches and control algorithms for prosthetics and robotic applications.

## Background & Summary

Active upper-limb prostheses rely on the collection of diverse signals, typically through electromyography (EMG). These signals are subsequently processed to understand user’s intention and control the device motors accordingly^1^. A critical step of this process is thus to evaluate which type (or combination) of signals is more appropriate for device control, trading off system complexity and computational cost with accuracy, robustness and redundancy. This is of pivotal importance especially for multi degrees of freedom (DOFs) devices. Several input sources have been proposed, either alone or in combination (e.g., by means of sensor fusion^2,3^) but a systematic comparison of approaches relying on different combinations of input signals is missing. For such a comparison, there is the need for a comprehensive dataset containing electromyographic signals (both low and high density), kinematic and tactile data of the whole upper body during the execution of simple and complex movements. The list of the publicly available datasets in Table 1 shows that neither of those fulfils all the requirements to support a thorough exploration of the potentially available input sources useful to deploy a safe and reliable prosthetic control.

**Table 1:** Available datasets.

Dataset	High Density-EMG	Low Density-EMG	Kinematic	Tactile Glove
Atzori *et al*.^15^, 2012	✗	✔	✔	✗
Atzori *et al*.^16^, 2014	✗	✔	✔	✔
Saudabayev *et al*.^17^, 2018	✗	✗	✔	✗
Jarque-Bou *et al*.^18^, 2019	✗	✔	✔	✗
Jarque-Bou *et al*.^19^, 2019	✗	✗	✔	✗
Matran *et al*.^20^, 2019	✔	✔	✔	✗
Jarque-Bou *et al*.^21^, 2020	✗	✔	✔	✗
Han *et al*.^22^, 2020^*^	✗	✔	✔	✗
Malesevic *et al*.^23^, 2021	✔	✗	✗	✔
Furmanek *et al*.^24^, 2022	✗	✔	✔	✗
Hernández *et al*.^25^, 2023	✗	✔	✔	✗
Mastinu *et al*.^26^, 2024^†^	✗	✗	✔	✗
Di Domenico *et al*.^27^, 2024	✔	✗	✗	✗
Di Domenico *et al*.^4^, 2024	✔	✔	✔	✔

*Additional types: eye-view and hand-view cameras.

^†^Additional types: radar sensors, proximity sensors and load cells.

To fill this gap, we built a dataset^4^ by collecting experimental data from 10 healthy subjects. The participants were asked to perform different upper-limb movements, including reaching and grasping movements (Table 2) with the right hand and arm, while collecting EMG (both in low- and high-density configurations), kinematic data (with motion capture system and a sensorized glove) and touch information (with a sensorized glove).

**Table 2:** Tasks name and description of movements starting from rest position.

	Task name	Abbreviation	Description
1	Hand opening	HO	Extend the fingers
2	Hand closing	HC	Flex the fingers
3	Wrist pronation	WP	Palm facing down
4	Wrist supination	WS	Palm facing up
5	Wrist flexion	WF	Bend the hand so that the palm faces the forearm
6	Wrist extension	WE	Lift the hand backwards so that the back of the hand faces the forearm
7	Cylindrical grasp	Cyl	Grasp a glass having a cylindrical shape
8	Spherical grasp	Sph	Grasp a tennis ball
9	Tri-digital grasp	Trid	Grasp a ping-pong ball with the first three fingers
10	Thumb opposition	Thumb	Thumb abduction
11	Frontal Reaching	FroRea	Move the hand toward the centre of a table
12	Reaching cylindrical	ReaCyl	Approach and grasp the cylindrical glass
13	Reaching spherical	ReaSph	Approach and grasp the tennis ball
14	Pour	Pour	Approach, grasp and pour the glass
15	Screw	Screw	Approach, grasp with the first three fingers and screw a bottle
16	Eat Fruit	EatFruit	Approach, grasp and move the tennis ball toward the mouth

Investigation of different types of signals (e.g., EMG, kinematics) during object manipulation from healthy individuals is pivotal for understanding the relationship between muscular activation and joint kinematics, which will drive the development of new control strategies for active prosthetics. Moreover, because of the pivotal role of sensory feedback during object manipulation, tactile data paves the way to studies involving feedback restoration strategies.

The added value of our dataset thus consists in the wide variety of signals that can be used to characterize upper-limb movement. For example, a subset of our dataset can be used for investigating a specific feature of motor control^5^, to test novel algorithms for pattern recognition^6^ or to investigate which finger and palm zones are involved in specific grasping tasks^7^.

Our dataset could improve traditional EMG-based control: while kinematics can be useful to correct EMG-based information during the execution of movements, tactile information can be helpful in increasing the information content during object manipulation and exploration. While being collected with the primary aim of improving the control techniques of upper-limb prostheses, the relevance of this dataset goes well beyond this scope, encompassing the study of motor control during voluntary movement of the upper-limb, up to the evaluation of different pattern recognition algorithms for decoding the generated movement from different data sources.

## Methods

### Participants

The recruited subjects were 10 healthy individuals (4 females and 6 males), right-handed without known neuromuscular impairments and aged between 24 and 36 years (age: 30.3 ± 4.0 years, weight: 69.5 ± 15.4 kg, height: 172.4 ± 8.0 cm, respectively mean ± standard deviation). Study participants were recruited among IIT graduate students and personnel.

Written informed consent was obtained from participants before data collection. To ensure data privacy and confidentiality, each participant was assigned a unique ID. The association table linking participant identities to their ID numbers was encrypted and securely stored and is not shared with the dataset.

All experiments were conducted in line with the Declaration of Helsinki and approved by the local ethical committee (CER Liguria Ref. 11554 of October 18, 2021).

### Experimental setup and data acquisition

**Fig. 1:**
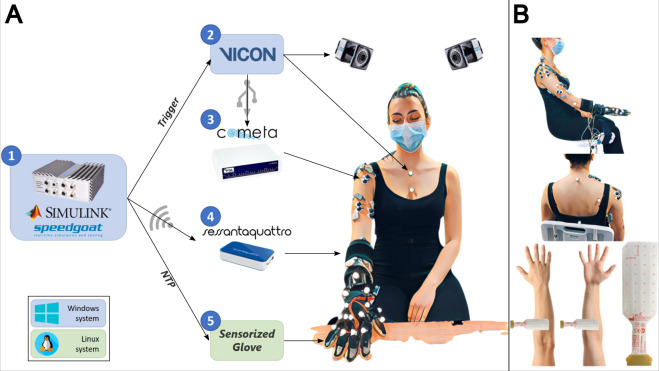
Experimental setup. (**A**) 1) Real Time target machine Speedgoat running the Simulink model. 2) Vicon motion capture system. 3) Cometa system bipolar EMG. 4) Sessantaquattro high-density EMG. 5) Sensorized glove from Bielefeld University. (**B**) Placement of the acquisition sensors: 23 reflective markers for the Vicon system, 10 bipolar EMG electrodes, 2 grids for HD-sEMG acquisition, and sensorized glove.

Figure 1 shows the devices used to record myoelectrical, biomechanical and tactile signals.

Myoelectric activity was measured with high-density and bipolar surface EMG.

High-density surface EMG (HD-sEMG) was recorded with the Sessantaquattro device (OT-Bioelettronica, Fig. 1A.4) using two pre-gelled adhesive monopolar grids of 32 electrodes (REF GR10MM0804, LOT GR0319, Fig. 1B), with the reference electrode placed on the ulnar styloid process. These portable grids were attached to the forearm of the subject proximally 5 cm below the olecranon. The first patch (electrodes 1–32) was placed over flexor muscles of the fingers (flexor digitorum superficialis, flexor carpi radialis, and ulnaris), while the second patch (electrodes 33–64) was placed on the extensor muscles of the fingers (extensor digitorum communis, extensor carpi radialis, and ulnaris). Thus, the first 32 channels refer to the flexor muscles activation, whilst the last 32 channels refer to the extensor muscles contraction. The position of the patches was decided as to cover the entire circumference of the forearm. The enclosure of the Sessantaquattro device was fixed on the leg of the subject through an elastic band. Data was collected with 2 kHz sampling frequency, 16 bits resolution and a full-scale input range of 18.75 mV peak to peak. The DC component was internally removed via a first order high-pass filter, with cut-off frequency of 10.5 Hz. The channels numbers, their abbreviations, and the description of the physical placements of the HD-EMG grids are reported in Supplementary Table 1.

Bipolar EMG was collected wirelessly with the Wave Plus system (Cometa, Fig. 1A.3). The myoelectric activity was measured through 10 bipolar pre-gelled adhesive electrodes placed on the following muscles: Upper Trapezius, Anterior Deltoid, Middle Deltoid, Posterior Deltoid, Long Head of Biceps, Short Head of Biceps, Lateral Head of Triceps, Medial Head of Triceps, Brachioradialis and Pronator Teres. Data was collected with a sampling rate of 2 kHz, 16 bits resolution and a full-scale input range of 5 mV peak to peak. The DC component was internally removed via a first order high-pass filter, with cut-off frequency of 10 Hz. Channel numbers, their abbreviations, and the description of the physical placements of the bipolar EMG electrodes are reported in Supplementary Table 2.

The Wave Plus system was connected via USB to the PC running the Vicon Nexus acquisition software, allowing the synchronization of the data coming from the Cometa (bipolar EMG) device with the kinematics data measured through the Vicon system.

Kinematic data was recorded with motion capture technology by MoCap (Vicon, Fig. 1A.2) using 10 infrared cameras and 23 reflective markers. This system allows to retrieve joint angles starting from markers trajectories using kinematic models. The reflective markers for the motion capturing system were attached on bony eminences of the shoulder, elbow, wrist, knuckles, and phalanges, according to the Vicon Plug-in Gait model (Vicon, Oxford, UK) and RHand model (Vicon Bodybuilder models), as depicted in Fig. 1. These signals were sampled at 100 Hz. The anatomical joints’ numbers, their abbreviations, and the description of the movements are reported in Supplementary Table 3. The Vicon Nexus software was used to retrieve anatomical joint angles from the markers’ trajectories. The two Vicon models (i.e., *Plug-in-Gait* and *RHand Bodybuilder*) were used to compute the joint angles of the whole upper-arm and joints of the fingers, respectively. These Vicon models define the rigid body segments linking two consecutive markers on the body. Then, a standard inverse kinematics is applied through the Vicon Nexus software to retrieve the relative anatomical joint angles of the whole upper arm starting from the markers’ trajectories. Each of the joints enlisted in Supplementary Table 3 has the representation of the rotation around the three Cartesian axes fixed to the corresponding joint. Therefore, a hinge-joint (e.g., elbow) will have only the rotation around its principal axis and the other two rotations will be close to zero. Conversely, a ball-and-socket joint (e.g., shoulder) will have each rotation axes dedicated to a specific movement (i.e., X: flexion/extension, Y: abduction/adduction, Z: internal rotation). The *RHand Bodybuilder* model computes the joint angles for the right hand and fingers. As output of this model, each finger contains both *absolute* (Abs) and *projected* (Proj) angles. Specifically, the Abs angles are calculated based on single vector representations of each finger segment where it is modelled as a hinge joint. In addition, the Proj angles represent two degrees of freedom (flex/extension and ab/adduction) of the metacarpophalangeal (MCP) joint. Overall, we collected the MCP joint for all the fingers (i.e., RThumbJ2, RIndexJ1, RMiddleJ1, RRingJ1, RPinkieJ1) and the scaphotrapeziotrapezoid (STT) joint only for the thumb (i.e., RThumbJ1).

**Fig. 2:**
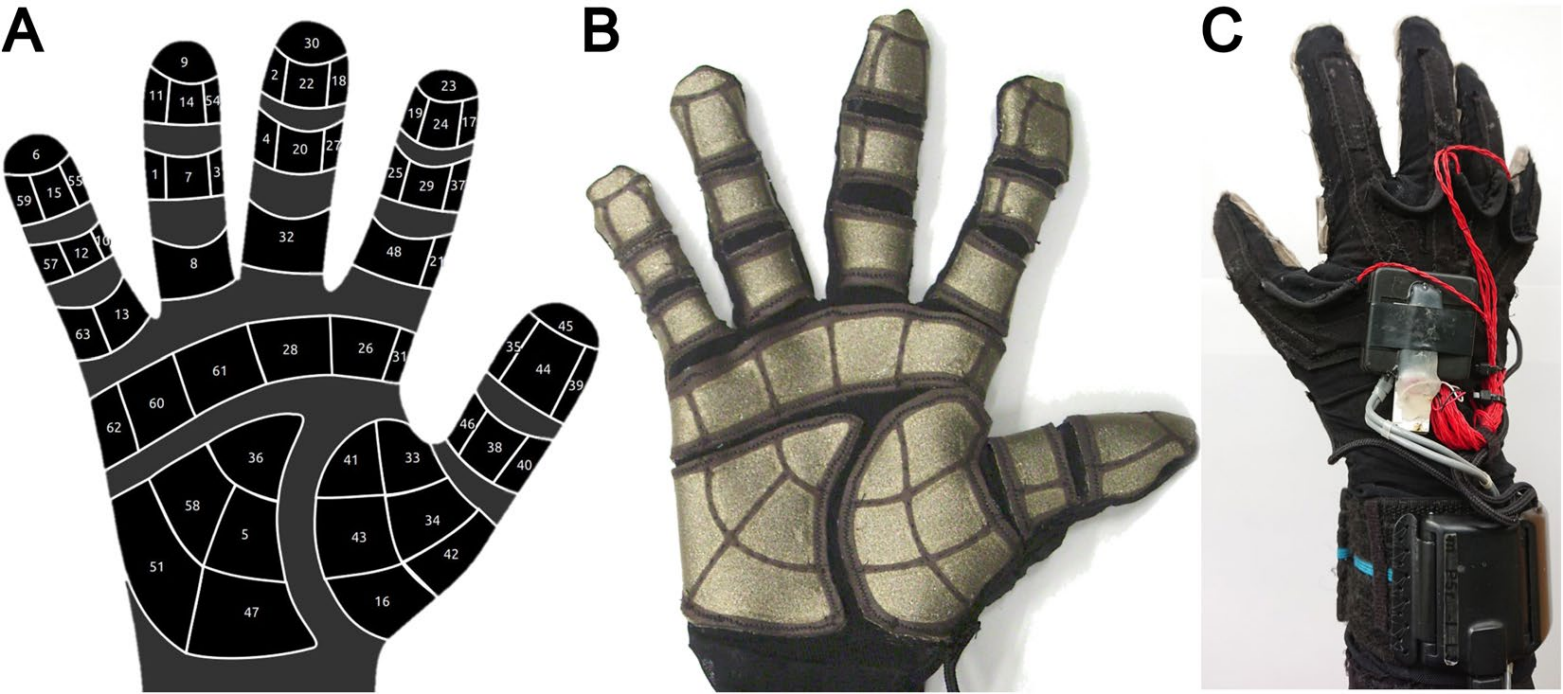
Sensorized glove. (**A**) schematic representation of the taxels (tactile pixels) and the corresponding numbers. (**B**) Front view of the glove palm with its 58 taxels. (**C**) Back view of the glove with the embedded electronics.

We additionally used a sensorized glove (Fig. 2) developed by the Bielefeld University^8^ to detect both fingers bending (kinematic data) and the exchanged forces during grasping (tactile data). We refer to it either as *cyberglove* for the collection of kinematic data, or *tactileglove* for the collection of touch data. The glove is connected to another computer running Linux OS (Ubuntu 20.04), communicates over ROS Melodic^9^, and is sampled at 100 Hz. ROS provides a communication protocol widely used in robot operation, which embeds sensor communication and data dumping^9^.

The kinematic data is measured by the CyberGlove I from CyberGlove Systems with no modifications. The glove measures the Metacarpophalangeal (MCP), and Proximal Interphalangeal Joints (PIP) of the index, medium, ring and pinkie fingers. The Distal Interphalangeal Joints (DIP) joint is usually assumed to have the same configuration as the PIP because they are a tendon coupled system. On the thumb, the MCP and the Interphalangeal Joint (IP) are measured. The glove additionally measures the relative finger abduction and the thumb rotation. Moreover, the wrist movements are captured. Specifically, the Wrist Pitch corresponds to the flexion/extension movement, whereas the Wrist Yaw represents the radial/ulnar deviation. The value of the joint angle is derived from an indirect measurement of the sensor deformation due to the bending of the finger. The scaling factor required to translate the sensor elongation to the joint angle must be tuned independently for each subject. This can be done either using the subject’s hand shape and size, to reflect the correct kinematic, or using the data of the Vicon motion capture system as second independent measurement.

The tactile data is measured by a soft and flexible piezoresistive pressure sensor integrated at the palm and at the inside of the fingers by the Bielefeld University^8^ as part of the WearHap EU-project (Grant agreement ID: 601165). The glove sits tight and forms the shape of the fingers with minimal impact on task execution. The sensor readings are related to the applied pressure on the single tactile sensor pad (taxel), because the material changes its resistance due to deformation. Using a pullup resistor and an ADC, the voltage change is converted and communicated through USB. The glove is not calibrated, meaning that we cannot relate the measurements to the applied force in terms of Newton, or pressure in Pascal, but the response of different taxels across the device is similar and thus relative comparison is possible. The tactile readings offer a measurement of the pressure applied over time and between different conditions, giving further insights on contact surface area and force distribution during manipulation. The glove has overall 58 taxels (Fig. 2A,B), 17 are located on the palm in three groups, while the others are located on the fingers: index and pinky finger have 9 sensors each, middle and ring 8, and the thumb 7. The sensors 9, 11, and 14 at the tip of the ring fingers do not provide reliable measurements due to a hardware issue, these taxels were thus discarded from data validation (see Fig. 9).

Although the sensorized glove added an extra layer, this did not affect object manipulation. Indeed, we selected tasks and objects to ensure that hand movements with the glove remained consistent with those without it, as verbally confirmed by the study participants. This was further supported by the absence of reported discomfort or difficulty, and no objects slipped or were dropped during any session.

Dataset acquisition required three computers. The ROS infrastructure for the *tactile glove* was running on a dedicated machine in Linux. The other machines were configured to run in Windows: one of the two machines was used to run the MoCap software, while the other hosted a custom-made Matlab GUI (The Mathworks, Natick USA) for streaming the data of the Sessantaquattro system. This latter machine was also used for time synchronization between all the devices. The Simulink model for time synchronization and HD-sEMG recordings was deployed on the Real-Time Target machine (Speedgoat Inc., Fig. 1A.1), allowing the synchronization between all the connected devices, by means of a hardware voltage trigger signal sent to both the Sessantaquattro and Vicon systems. Moreover, the clock between the Linux and the Window machine were synchronized using the Network Time Protocol (NTP).

All experimental sessions were video recorded. This data is not provided but is available upon request.

### Experimental protocol

The recruited subjects were required to perform different tasks involving simple movements (i.e., single DoFs), reach, grasp or even manipulation of objects with different shapes (e.g., cylinder, sphere). In total the dataset contains 16 different tasks with 10 repetitions each.

The tasks are enlisted in Table 2, and ranged from simple to complex tasks starting from a rest position with the elbow bended at 90° and the wrist aligned with the forearm. The selected tasks were chosen in order to involve the entire upper-limb during reach and grasp movements that correspond to highly common daily-life gestures.

**Fig. 3:**
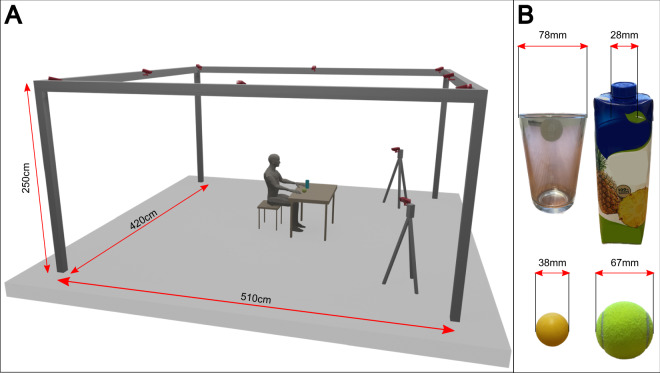
Acquisition environment and grasped objects. (**A**) Representation of the 10 infrared Vicon Cameras (highlighted in red) pointing toward the acquisition volume where the subjects performed the experiment. (**B**) Dimensions of the grasped objects: glass (cylindrical grasp), bottle cap (screw), table tennis ball (tridigital grasp) and tennis ball (spherical grasp).

Simple movements consisted in hand opening/closing (HO/HC), wrist pronation/supination (WP/WS), wrist flexion/extension (WF/WE), thumb abduction (Thumb), grasping an object (Fig. 3B) that could be cylindrical (Cyl), spherical (Sph), or requiring tri-digit grasping (Trid). During the complex movements, the subjects were asked to perform unconstrained reach and grasp movements sequentially, e.g. frontal (FroRea), cylindrical (ReaCyl) or spherical reach (ReaSph), or even to manipulate the grasped objects, e.g. approach, grasp and pour the glass (Pour), approach, grasp with the first three fingers and screw a bottle cap (Screw), or approach, grasp and move the tennis ball toward the mouth (EatFruit). Since no timing was imposed to the subjects, we asked them to perform all the repetitions roughly at a fixed velocity. We discarded time constraints to leave the subjects free to make the movements in the most natural way. After each task, subjects rested for 1 minute to prevent fatigue, thus the total experimental acquisition last approximately 1 hour.

## Data Records

### Data conversion to BIDS standard

**Fig. 4:**
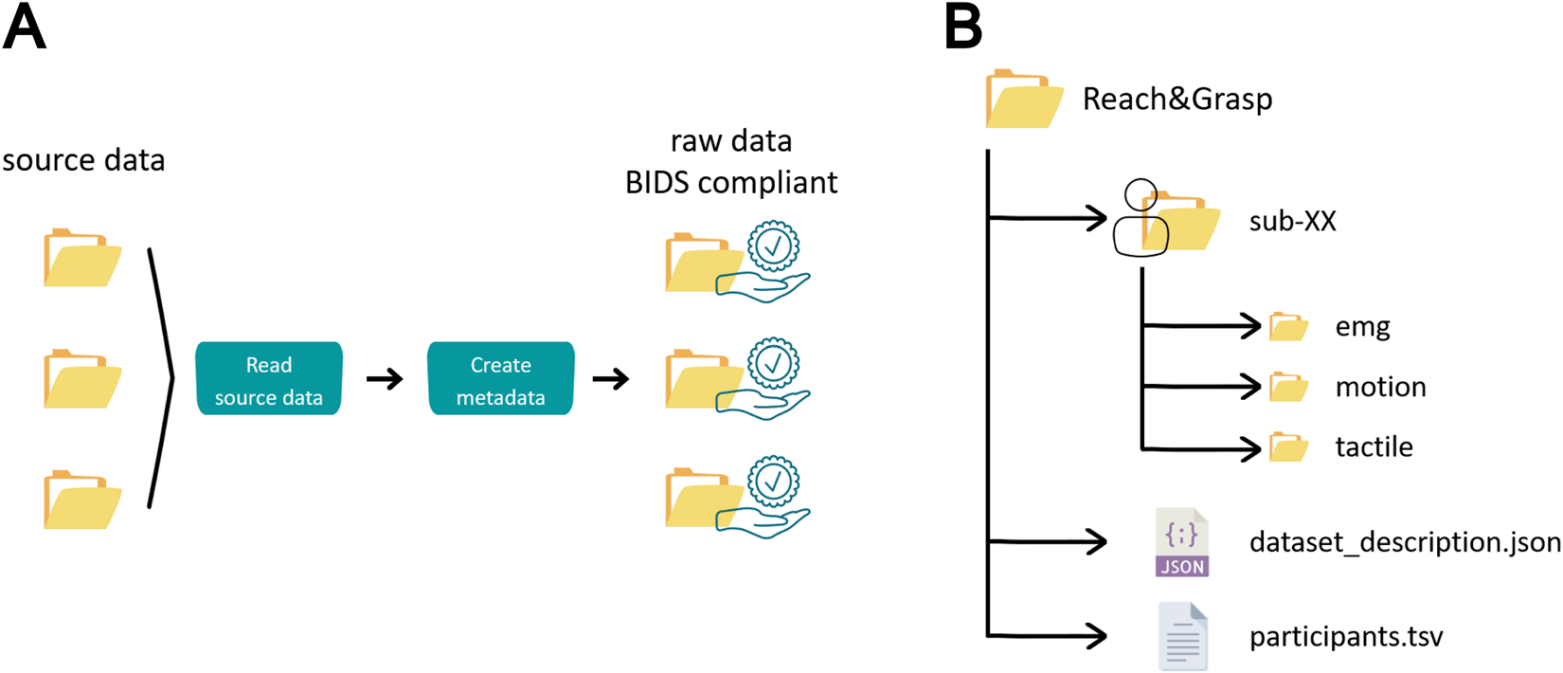
Folders organization and structure. (**A**) General overview of the data conversion pipeline for data standarization. (**B**) Structure of the root folder of the dataset containing the BIDS compliant files.

The Reach&Grasp dataset is freely available on IIT Dataverse^4^. The data was structured according to the Brain Image Data Structure (BIDS) standard^10^, for which a dedicated data conversion pipeline was created. A specialized data conversion pipeline was developed, which systematically organized the data into a hierarchical filesystem structure consistent with BIDS conventions. This automated pipeline initialized the creation of folders and files in accordance with the BIDS framework for each participant, as illustrated in Fig. 4A.

The data conversion pipeline is based on the function *ft_data2bids* from Fieldtrip^11^, which is a Matlab open-source software for electroencephalography analysis. This function was adapted to convert the collected data (*source data* in Fig. 4A) to BIDS-compliant formats, and to create their appropriate metadata. The data conversion pipeline consists of a master script that reads a root folder named *‘source data’* and populates a BIDS compliant data structure according to the number of subjects and data types present in the source folder. Then it creates metadata according to each data types, calling a customized version of *ft_data2bids* function.

Because the BIDS format was originally conceived for neuroimaging data (e.g., MEG, EEG), the function *ft_data2bids* had to be adapted for our specific datatypes: EMG, motion and tactile data. Each data type was converted into a BIDS-compliant format following draft guidelines produced by the working groups of the BIDS community.

Due to the large size of source data, we decided to share the raw BIDS compliant dataset only. Thus, we are not providing the code used to convert source data into BIDS compliant raw data, which can be shared upon request.

The central directory (‘*Reach&Grasp*’ in Fig. 4B) contains the required BIDS compliant folder for each subject, along with its metadata. Subjects’ folders are named as *sub-XX*, were *XX* indicates subject number (e.g., *sub-01*, *sub-02*, …, *sub-10*). Within each subject folder, there are 3 subfolders named as *emg*, *motion*, and *tactile*, for EMG, kinematic and touch data, respectively (Fig. 4B). Within each folder there are 3 different files related to each of the 16 movements (listed in Table 2), which are:*.csv* file containing raw data,*.json* file containing metadata of the specific recording modality, and *.tsv* file containing channels description.

Regarding file naming, *.csv* and *.json* files are named as *sub-XX_task-YY_acq-ZZ_FF*, where *XX* indicates subject number, *YY* indicates movement label (see column ‘Abbreviation’ in Table 2), ZZ indicates the acquisition system (i.e., *cometa* or *sessantaquattro* for EMG data, *vicon* or *cyberglove* for kinematic data, and *tactileglove* for touch data), and *FF* denotes the containing folder, which also corresponds to the data type (i.e., *emg*, *motion*, and *tactile*).

The *.tsv* file is named as *sub-XX_task-YY_acq-ZZ_channels*, which is the same as the other files with the exception that the suffix *channels* takes the place of folder name (*FF*).

File *.csv* contains timestamps in the first column, while other columns contain single channels data. This file has no header and channels names are enlisted in the *.tsv* file in the same order.

*The .tsv* file also contains other information, as detailed in Supplementary Table 6.

Meta data is contained in the*.json* files, which provide several information as detailed in Supplementary Table 7.

### Data repository

IIT Dataverse is the institutional research data repository of Istituto Italiano di Tecnologia, for both preservation and sharing of research datasets. It is based on the Dataverse software, developed at Harvard University. Dataverse assigns persistent DOIs to all data uploads for findability and it is accessible through standard HTTPS protocol. IIT Dataverse is indexed in OpenAIRE Explore and registered in re3data.org. Moreover, it provides APIs to search and access datasets, including a SWORD API. The uploaded data includes citation metadata, optional and customizable domain-specific metadata (e.g., for Life Sciences), and file-level metadata. Metadata can be exported in different standard formats (DataCite 4, OpenAIRE, JSON, JSON-LD, OAI, etc.) for maximal interoperability. Dataverse ensures reusability of datasets, by supporting open licenses, like Creative Commons licenses, and offers the possibility to customize specific data usage agreements. IIT Research Data Management service oversees dataset publication, by providing basic data curation to ensure dataset quality and FAIRness^12^ (Findable, Accessible, Interoperable, and Reusable).

## Technical Validation

### Validation of EMG data

**Fig. 5:**
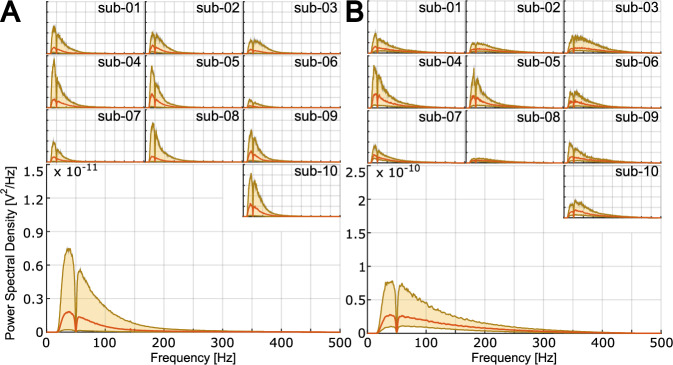
Spectral analysis of high density (**A**) and bipolar (**B**) EMG data. Top plots represent single subjects’ PSD, while the larger bottom plots represent the average across subjects. Red lines represent the mean PSD while the lower and upper border of the shaded are represent the 25^th^ and 75^th^ percentile respectively. For each panel, the axes limit of the single subject are the same of the corresponding plot representing the average across subjects.

To verify the quality of the EMG data for both bipolar and high-density signals, we performed a frequency analysis. Since the spectrum of the EMG signals ranges between 10 Hz and 500Hz^13^, we first applied a 4^th^ order bandpass filter (cut-off frequencies: 10 Hz and 500 Hz), we then used a 6^th^ order notch filter (cut-off frequency: 50 Hz) to attenuate the power-line interference. After filtering the EMG data, we computed Power Spectral Density (PSD) via the Welch’s method^14^, to estimate the spectrum of the acquired signals. The PSD for the HD-sEMG and bipolar EMG are graphically represented in Fig. 5A,B, respectively.

### Validation of Kinematic data

For evaluation of kinematic data from the Vicon system and the Cyberglove, we considered the angle variation of the joints involved during each task and movement time. Hand and wrist kinematics were analysed with both types of data, while kinematics of elbow and shoulder are only supported by Vicon data.

For hand opening and closing (HO/HC) we observed the joints related to each finger in flexion/extension: Metacarpophalangeal (T_MCP_) and Scaphotrapeziotrapeziodal joint of Thumb (T_STT_), Metacarpophalangeal joint of Index finger (I_MCP_), Metacarpophalangeal joint of Middle finger (M_MCP_), Metacarpophalangeal joint of Ring finger (R_MCP_), Metacarpophalangeal joint of Pinkie finger (P_MCP_), wrist, elbow and shoulder in flexion/extension (W_FE_, E_FE_, S_FE_) and wrist in pronation/supination (W_PS_). W_FE_ and W_PS_ were calculated from Vicon system data, while the Cyberglove allowed calculation of wrist pitch (W_PITCH_) and yaw (W_YAW_).

**Fig. 6:**
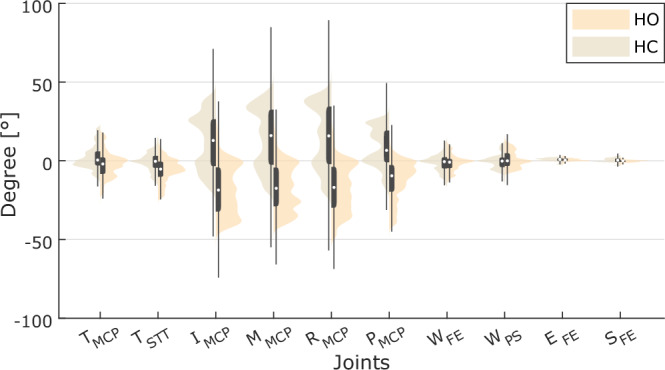
Distribution of joints kinematic from Vicon data in hand opening and closing (HO/HC). Violin plot of angle variation in different tasks. Superimposed in dark grey are boxplots describing the median value (white dot), 25^th^ and 75^th^ percentiles (extremes of the box), and full data range (extremes of the thin grey line) of the distributions.

As depicted in Fig. 6, we observe little angle variation for elbow and shoulder joints since the subjects were asked to keep their arm in a fixed position, while the hand was moving. Conversely, higher angle variation was found for index, third, ring and pinkie fingers, because they are mostly involved in HO/HC movements. The bimodal distribution of angle variation in each joint is resulting from considering both HO/HC movements and the rest in between (Supplementary Figures 1, 2).

**Fig. 7:**
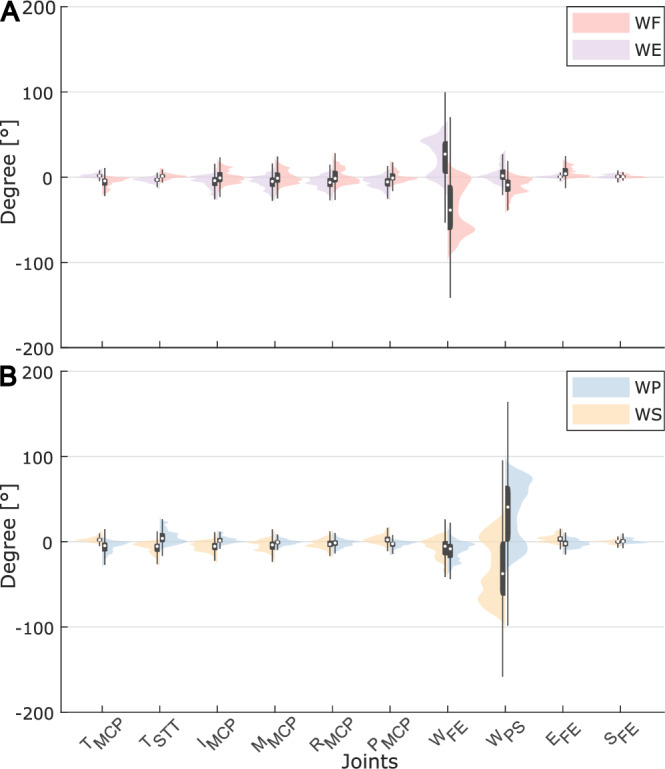
Distribution of joints kinematic from Vicon data in (**A**) wrist flexion and extension (WF/WE) and (**B**) pronation/supination (WP/WS). Violin plot of angle variation in different tasks. Superimposed in grey are boxplots describing the median value (white dot), 25^th^ and 75^th^ percentiles (extremes of the box), and full data range (extremes of the thin grey line) of the distributions.

For wrist movements, i.e., wrist flexion/extension (WF/WE) and pronation/supination (WP/WS), we considered only wrist, elbow and shoulder in flexion/extension (W_FE_, E_FE_, S_FE_) and wrist in pronation/supination (W_PS_); wrist movements from Cyberglove were considered in terms of pitch (W_PITCH_) and yaw (W_YAW_) (Supplementary Figures 1, 2). As reported in Fig. 7, elbow and shoulder show little angle variation because the subjects were asked to keep their arm in a fixed position, while the wrist was moving. As expected, higher angle variation was found for the wrist joint. The bimodal distribution of angle variation in each joint is resulting from considering WF/WE (Fig. 7A) and WP/WS (Fig. 7B) movements and the rest in between.

**Fig. 8:**
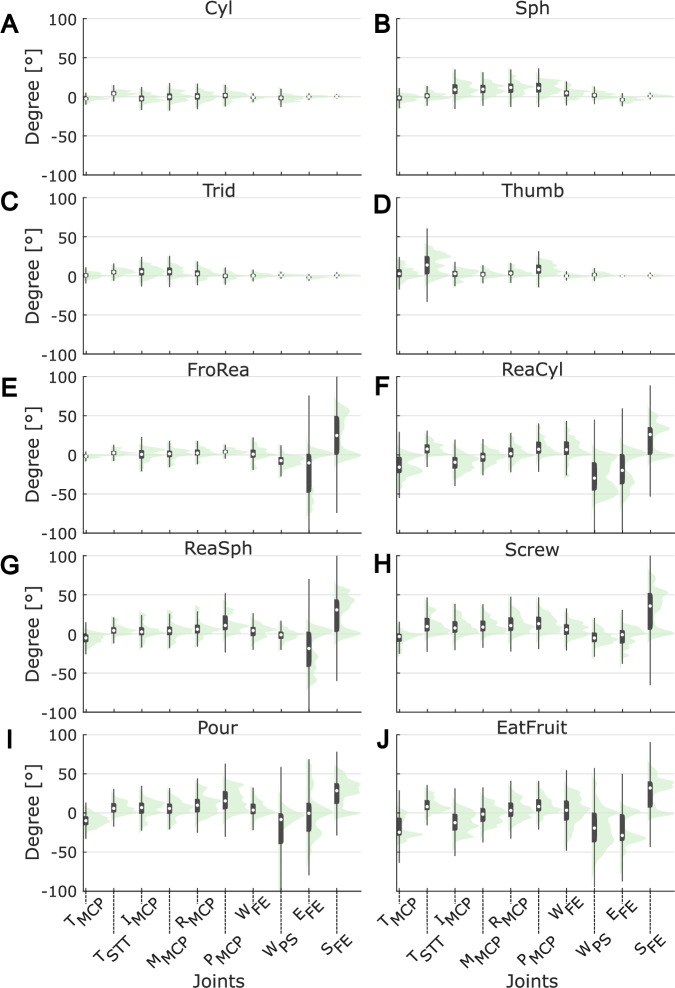
Distribution of joints kinematic from Vicon data for reaching and grasping movements. The represented tasks are: Cylindrical grasp (**A**), Spherical grasp (**B**), Tridigital grasp (**C**), Thumb opposition (**D**), Frontal reaching (**E**), Reaching and Cylindrical grasp (**F**), Reaching and Spherical grasp (**G**), Screw (**H**), Pour (**I**), EatFruit (**J**). Violin plot of angle variation in different tasks. Superimposed in grey are boxplots describing the median value (white dot), 25^th^ and 75^th^ percentiles (extremes of the box), and full data range (extremes of the thin grey line) of the distributions.

Angle variations during movements of all the other tasks are reported in Fig. 8 and Supplementary Figure 3, considering all the hand, wrist, elbow and shoulder joints. Cylindrical grasp (Fig. 8A) requires less finger joints movements than spherical grasp (Fig. 8B), having the cylindrical object a greater diameter (~10 mm) than the spherical object one (Fig. 3B). The tridigital grasp involves movements of all the fingers, but mainly the thumb, index and middle ones; this trend has been confirmed in our data (Fig. 8C). The thumb opposition mainly produced movements in the thumb joints (Fig. 8D). We noted that, in this case, also the pinkie was moving as we requested the subjects to touch their palm with the thumb as close as possible to pinkie, it is thus possible that subject slightly flexed their pinkie to facilitate the task. As expected, in these movements involving only grasping, the elbow and shoulder joints exhibit low angle variation (Fig. 8A–D), conversely for reaching and grasping movements their angle variation is wide (Fig. 8E–J). Frontal reach movement mainly activates the joint of the elbow and shoulder, whereas the fingers and wrist joints are minimally recruited (Fig. 8E). Figure 8F–H show reaching with cylindrical, spherical and tridigital grasping, respectively, thus the joints involved are both those involved in simple grasping (Fig. 8A–C) and those involved in frontal reaching (Fig. 8E). In the Pour and EatFruit movements, all the considered joints were involved (Fig. 8I,J). The bimodal distribution of angle variation in each joint is resulting from considering both go-and-back sub-movements and the rest in between.

### Validation of tactile data

The tactile data acquired does not directly correspond to force or pressure. Rather, it captures measurements of electrical resistance within the piezoresistive material present in the inner lining of the glove, which is impacted by a combination of contact geometry and contact force. The measurements shed insights on the distribution of force across the palm and finger surface allowing to picture the contact properties.

**Fig. 9:**
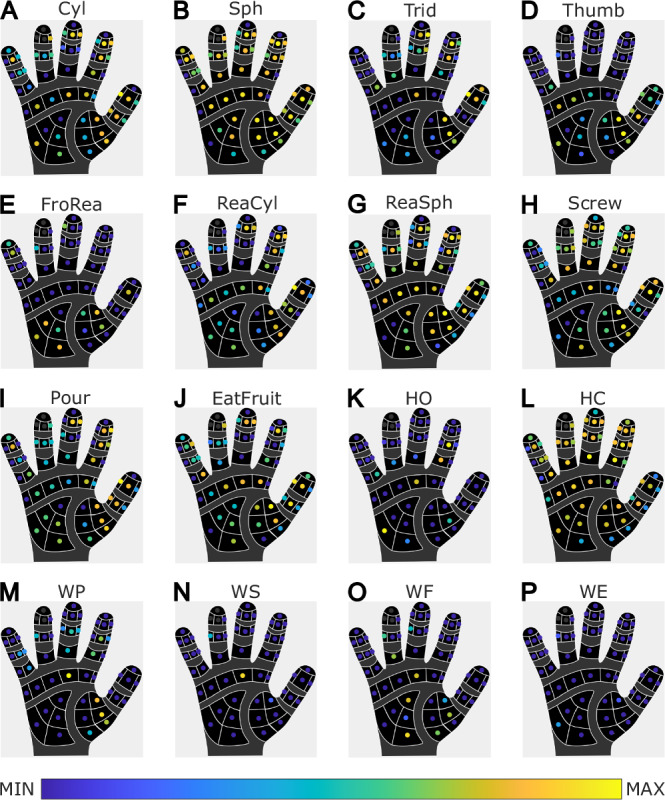
The average taxel activity is displayed, log-scaled for clarity. Tasks with object interaction show notably higher sensor readings. Even in tasks without direct object interaction, there are readings above zero due to material deformation (see main text for explanation). Different grasp strategies like tridigit and spherical grasp or cylindrical and spherical grasp are distinguishable. The taxels rdd (n. 9), rdo (n. 11) and rdm (n. 14) are reported in grey due to their malfunction.

Comparing tasks involving contact with objects yield notably higher sensor readings in comparison to tasks without contact. However, tasks without object contact still produce above zero sensor readings. This is caused by fabric deformation when folded or stretched. To validate the tactile data over all subjects and tasks, we calculated the mean taxel activity per task over all subjects. The results are shown in Fig. 9. excluding sensor 9, 11, and 14 due malfunctions.

During hand opening (Fig. 9K), little activity is detected by the Cyberglove, whereas during hand closing (Fig. 9L) high activity is detected over most of the palm. and all fingers with high activity due to the lateral pressure from the neighbouring fingers when forming a fist, taking out the most outer line of the pinkie and thumb. For wrist flexion/extension (Fig. 9O,P) and wrist supination (Fig. 9M,N) no major activity can be reported due to the lack of contacts, as in frontal reach (Fig. 9E). The only non-contact tasks which shows a significant trend in sensor readings is the thumb opposition task (Fig. 9D) due to the strong deformation of the fabric when it is folded over.

Looking into the tasks with object contact shows meaningful and interpretable results. Comparing the spherical and the cylindrical grasp (with or without reaching phase, Fig. 9A,B,F,G,I) shows subtle differences at the force distribution due to different grasping strategies. Using the spherical grasp distributes the force more evenly along the palm because all fingers fully enclose the object, in opposite to the cylindrical grasp which is more focused on the fingertips.

For tasks like tridigit (Fig. 9C,J) and screwing (Fig. 9H), most of the sensor activity was concentrated at the tips of the thumb, index, and middle finger, as expected, but we also noticed significant activity in the ring finger and palm. This makes sense because these tasks required a firm grip with all fingers to prevent interference, especially in the ring finger where the motion from the middle finger needs compensation. The pinkie finger could be more relaxed in these situations because the ring finger was already stabilizing the hand.

### Data segmentation

As described previously, each recorded task includes 10 repetitions. However, for further validation of the data (i.e., analysis of task duration – see next section), we segmented the data according to each repetition time interval by using as a reference the kinematics of the joint considered the most relevant for each task. To find the beginning and the end of each repetition, we adopted a criterion based on the derivative of the angular position, i.e., the angular velocity, by taking the locally highest and lowest value of the signal. When this segmentation was not possible (e.g., in absence of relevant finger movements during grasping tasks), we manually identified the rising and falling peaks, using the *ginput* Matlab function.

Only for subject #10 executing the Cyl task, neither automatic nor manual function could recognize the rising and falling peaks of kinematic data. Thus, for this case only, we used the signals from HD-sEMG to find the rising and falling peaks. We preferred to use kinematic data for data segmentation, because EMG-based segmentation would require the identification of a subject-specific threshold for movement detection, while kinematic-based segmentation is directly related to joint angle variation, which generally shows less inter-subject variability.

**Fig. 10:**
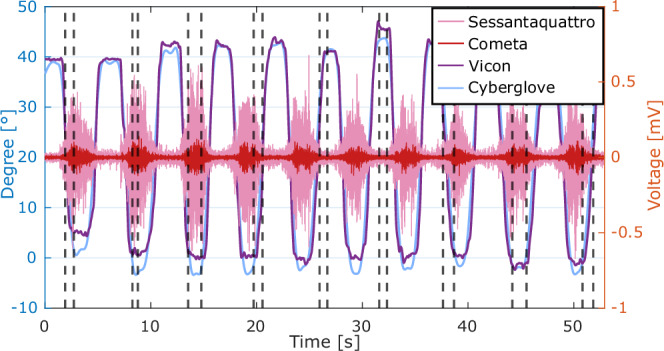
Data segmentation in a representative task repetition (Hand Opening) performed by one subject (sub-04). The plot shows Bipolar (Cometa, red signal), HD-sEMG (Sessantaquattro, pink signal) and glove (Cyberglove, light-blue signal) data segmentation starting from motion capture data (Vicon, violet profile). The black dashed vertical lines indicate the start and end times of each singular movement execution. Y-axis on the left (in blue) refers to the Vicon data, while Y- axis on the right (in orange) refers to both the Cometa and Sessantaquattro data.

Figure 10 represents data segmentation into 10 repetitions performed by one subject. Based on the function described above, we identified starts and ends of each singular movement execution (black dashed vertical lines in Fig. 10) according to the rising and falling peaks of the kinematic data obtained from the Vicon system (blue profile in Fig. 10). To validate the results, Fig. 10 also reports a single muscle channel of bipolar (Brachioradialis, red signal in Fig. 10) and high-density EMG data (placed on extensor muscles, pink signal in Fig. 10). As shown in the figure, the identified time events allow to separate the kinematic data into repetitions in which muscular activation is disconnected from resting in both bipolar and high-density EMG modalities.

### Tasks duration

**Fig. 11:**
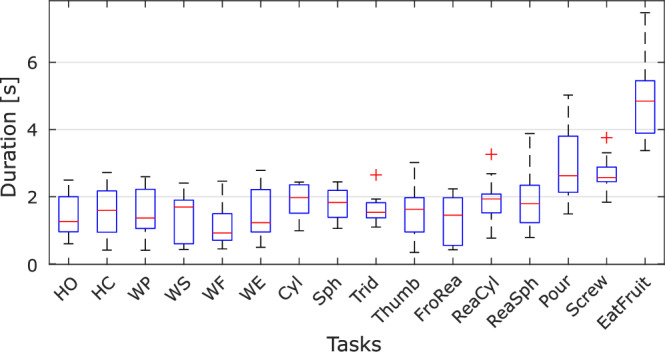
Distribution of task duration across subjects. The red line indicates the median, top and bottom limits of the boxplots respectively indicate the 25^th^ and 75^th^ percentile, the whiskers indicate the minimum and maximum value, and red crosses indicate outlier values (greater than 1.5 times the interquartile range of the data).

Data segmentation allowed us to evaluate the duration of each repetition of each task for each subject. Although we do not requested subjects to perform each task within a predefined time range, we observed similar task duration across subjects. Figure 11 shows the distribution of task duration (both go and back phases of the 10 repetitions of each requested movements) across subjects. As expected, simple movements required less time (1.53 ± 0.66 s) to be performed, while complex movements including reaching and grasping took on average 2.61 ± 1.45 s.

## Usage Notes

The instructions on how to download the dataset are described in the README of the GitHub repository. Please refer to “How to download the dataset” in the repository according to the operating system in use.

## Supplementary information

Supplementary Figures and Tables

## Data Availability

The Matlab code used to perform the presented data validation and to generate their respective figures was written using Matlab version R2022b and can be accessed at the following GitHub repository (https://github.com/DarioDiDomenico/SData_ReachGrasp). Following is a description of the provided functions. Within the GitHub repository, under the *code* directory there are several Matlab files (named as *Figure_X* and *SupplementaryFigure_X*, with X corresponding to the numbering of this article) available to generate all the figures, starting from the downloaded dataset. Please just run the code and follow the instructions. Moreover, we provide the *Events_Reach&Grasp.mat* file which contains the triggers of the movements as described in Data Segmentation paragraph.
