# Supplementary material for: Reach&Grasp: a multimodal dataset of the whole upper-limb during simple and complex movements
Source: Sci Data. 2025 Feb 7;12:233. doi: 10.1038/s41597-025-04552-5 (PMC11805991; doi:10.1038/s41597-025-04552-5)
Supplement: Supplementary file 1 — Supplementary Figures and Tables [file 41597_2025_4552_MOESM1_ESM.pdf]

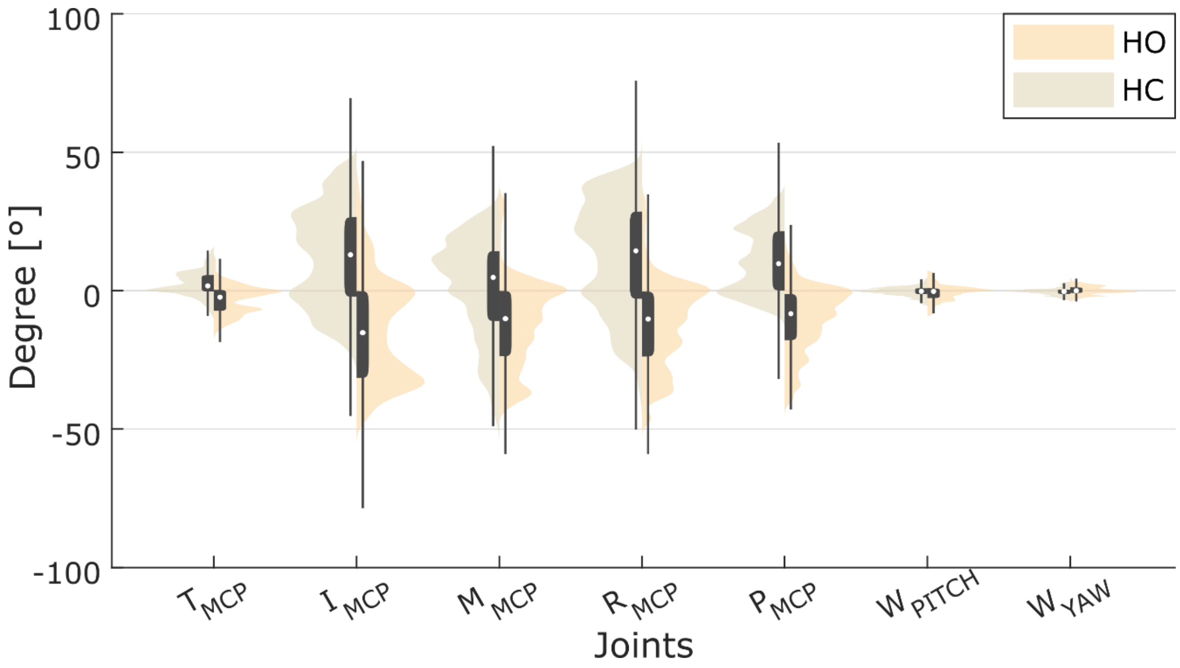

Supplementary Figure 1: Distribution of joints kinematic from Cyberglove data in hand opening and closing (HO/HC). Violin plot of angle variation in different tasks. Superimposed in dark grey are boxplots describing the median value (white dot), 25th and 75th percentiles (extremes of the box), and full data range (extremes of the thin grey line) of the distributions.

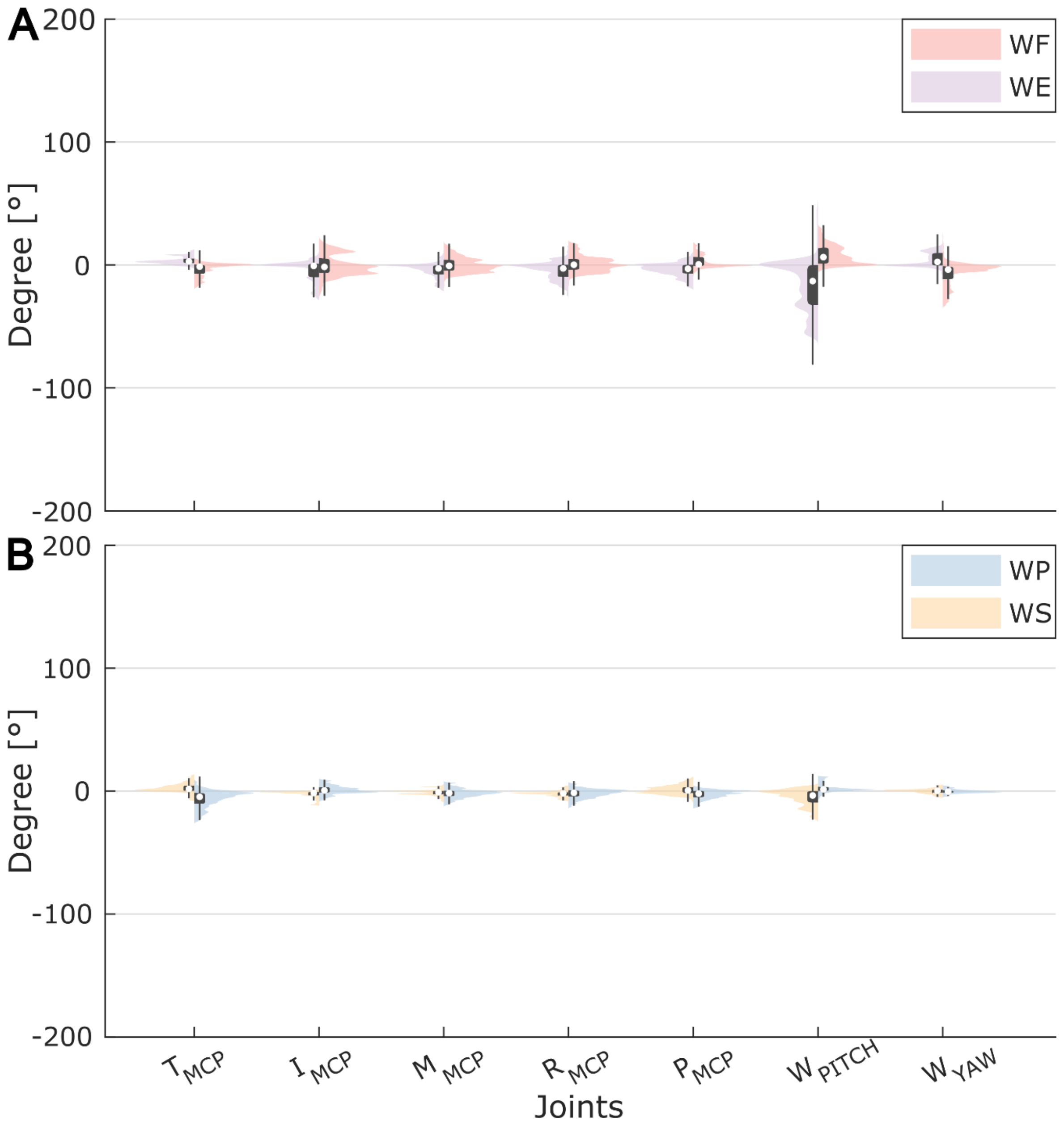

Supplementary Figure 2: Distribution of joints kinematic from Cyberglove data in A) wrist flexion and extension (WF/WE) and B) pronation/supination (WP/WS). Violin plot of angle variation in different tasks. Superimposed in grey are boxplots describing the median value (white dot), 25th and 75th percentiles (extremes of the box), and full data range (extremes of the thin grey line) of the distributions.

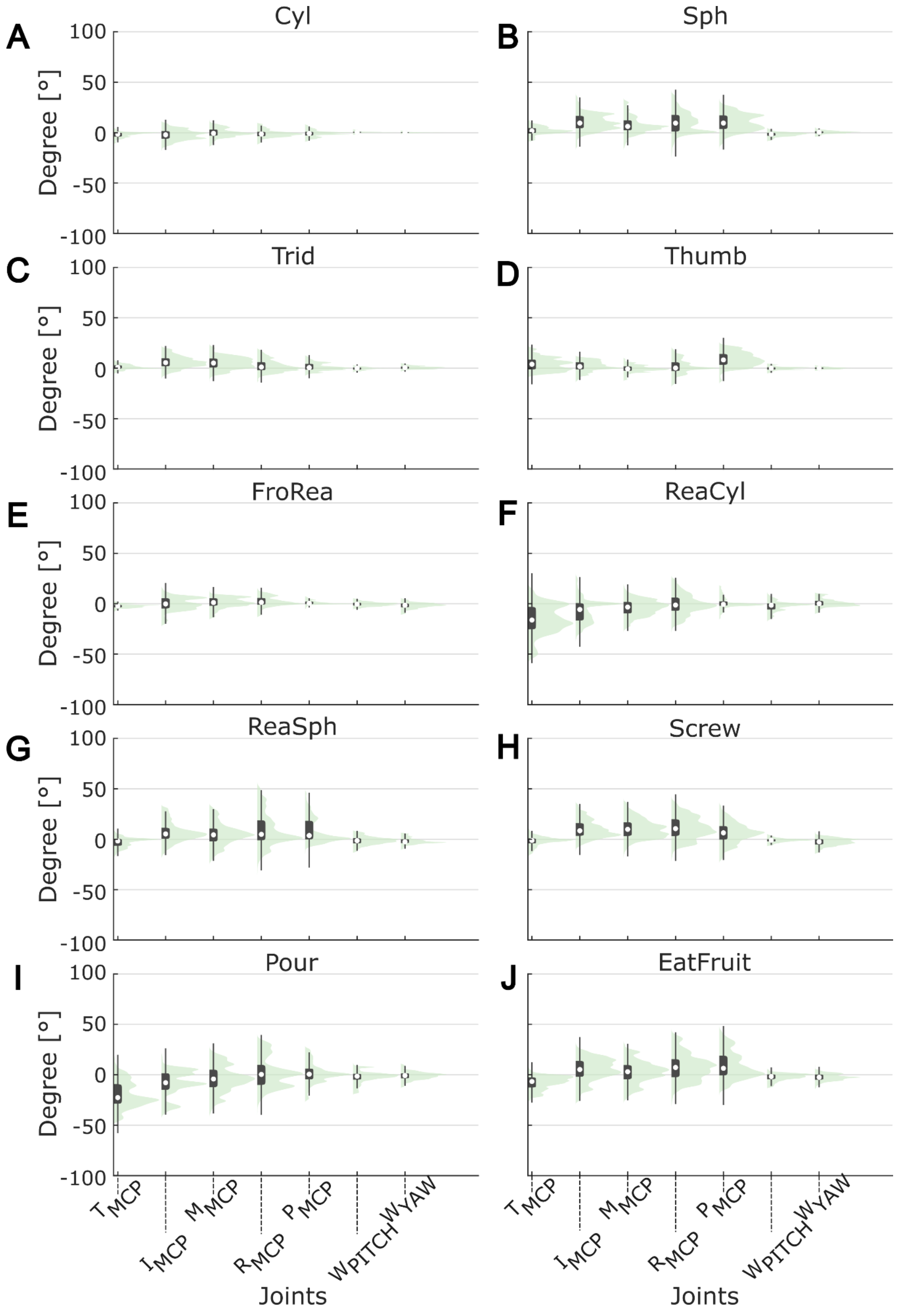
A
B
C
D
E
F
G
H
I
J
Cyl
Sph
Trid
Thumb
FroRea
ReaCyl
ReaSph
Screw
Pour
EatFruit
Degree [°]
100
50
0
-50
-100
T MCP
I MCP
M MCP
R MCP
P MCP
W PITCH
W YAW
Joints

*Supplementary Figure 3: Distribution of joints kinematic from Cyberglove data for reaching and grasping movements. The represented tasks are: Cylindrical grasp (**A**), Spherical grasp (**B**), Tridigital grasp (**C**), Thumb opposition (**D**), Frontal reaching (**E**), Reaching and Cylindrical grasp (**F**), Reaching and Spherical grasp (**G**), Screw (**H**), Pour (**I**), EatFruit (**J**). Violin plot of angle variation in different tasks. Superimposed in grey are boxplots describing the median value (white dot), 25th and 75th percentiles (extremes of the box), and full data range (extremes of the thin grey line) of the distributions.*

Supplementary Table 1: HD-EMG channels numbers, abbreviations, and description of the physical placement.

HD-EMG channel	**Abbreviation**	**Description**
1	flexion_01	Distal flexor muscle
2	flexion_02	Distal flexor muscle
…	…	…
31	flexion_31	Proximal flexor muscle
32	flexion_32	Proximal flexor muscle
33	extension_01	Distal extensor muscle
34	extension_02	Distal extensor muscle
…	…	…
63	extension_31	Proximal extensor muscle
64	extension_32	Proximal extensor muscle

Supplementary Table 2: Bipolar EMG channels numbers, abbreviations, and description of the physical placement.

EMG channel	**Abbreviation**	**Description**
1	Lat_Tric	Lateral Head of Triceps
2	Med_Tric	Medial head of Triceps
3	Long_Bic	Long Head of Biceps
4	Short_Bic	Short Head of Biceps
5	Ant_Delt	Anterior Deltoid
6	Midd_Delt	Middle Deltoid
7	Post_Delt	Posterior Deltoid
8	Upper_Trap	Upper Trapezius
9	Brachiorad	Brachioradialis
10	W_pronator	Pronator Teres

Supplementary Table 3: Anatomical joints' numbers, abbreviations, description of the movements.

Joints	Abbreviation	Description	
1	LThorax	LeftThorax	X: Backward tilt
			Y: Right tilt
			Z: Right rotation
2	RElbow	RightElbow	X: Flexion-Extension
			Y: -
			Z: -
3	RShoulder	RightShoulder	X: Flexion-Extension
			Y: Abduction
			Z: Internal rotation
4	RThorax	RightThorax	X: Backward tilt
			Y: Left tilt
			Z: Left rotation
5	RWrist	RigthWrist	X: Ulnar Deviation
			Y: Flexion-Extension
			Z: Pronation-Supination
6	RIndexJ1	RightIndex	Abs_X: Flexion-Extension
			Proj_Y: Flexion-Extension
			Proj_Z: Adduction-Abduction
7	RPinkieJ1	RightPinkie	Abs_X: Flexion-Extension
			Proj_Y: Flexion-Extension
			Proj_Z: Adduction-Abduction
8	RRingJ1	RightRing	Abs_X: Flexion-Extension
			Proj_Y: Flexion-Extension
			Proj_Z: Adduction-Abduction
9	RThirdJ1	RightThird	Abs_X: Flexion-Extension
			Proj_Y: Flexion-Extension
			Proj_Z: Adduction-Abduction
10	RThumbJ1	RightThumb	Abs_X: Flexion-Extension
			Proj_Y: Flexion-Extension
			Proj_Z: Adduction-Abduction
11	RThumbJ2	RightThumb	Abs_X: Flexion-Extension

Supplementary Table 4: Glove joints

Cyberglove Joints	**Abbreviation**	**Description**
1	ThumbRotate	Thumb rotation
2	ThumbMPJ	Thumb MCP
3	ThumbIJ	Thumb IP
4	ThumbAb	Thumb Abduction
5	IndexMPJ	Index MCP
6	IndexPIJ	Index PIP
7	MiddleMPJ	Middle MCP
8	MiddlePIJ	Middle PIP
9	MiddleIndexAb	Middle Index Abduction
10	RingMIJ	Ring MCP
11	RingPIJ	Ring PIP
12	RingMiddleAb	Ring Middle Abduction
13	PinkieMPJ	Pinkie MCP
14	PinkiePIJ	Pinkie PIP
15	PinkieRingAb	Pinkie Ring Abduction
16	PalmArch	Palm Arch
17	WristPitch	Wrist Pitch
18	WristYaw	Wrist Yaw

Supplementary Table 5: Glove taxels (n.a. means unused taxel)

Tactileglove taxels	**Abbreviation**	**Description**
1	rmo	RingMiddle
2	mdo	MiddleTip
3	rmi	RingMiddle
4	mmo	MiddleMiddle
5	pcim	PalmWrist
6	ldd	PinkyTip
7	rmm	RingMiddle
8	rp	RingPalm
9	rdd	RingTip
10	lmi	PinkyMiddle
11	rdo	RingTip
12	lmm	PinkyMiddle
13	lp	PinkyPalm
14	rdm	RingTip
15	ldm	PinkyTip
16	ptip	PalmThumb
17	idi	IndexTip
18	mdi	MiddleTip
19	ido	IndexTip
20	mmm	MiddleMiddle
21	ipi	IndexPalm
22	mdm	MiddleTip
23	idd	IndexTip
24	idm	IndexTip
25	imo	IndexMiddle
26	pdi	PalmFinger
27	mmi	MiddleMiddle
28	pdm	PalmFinger
29	imm	IndexMiddle
30	mdd	MiddleTip
31	pdii	PalmFinger
32	mp	MiddlePalm
33	ptod	PalmThumb
34	ptmd	PalmThumb
35	tdo	ThumbTip
36	pcid	PalmWrist
37	imi	IndexMiddle
38	tmm	ThumbPalm
39	tdi	ThumbTip
40	tmi	ThumbPalm
41	ptop	PalmThumb
42	ptid	PalmThumb
43	ptmp	PalmThumb
44	tdm	ThumbTip
45	tdd	ThumbTip
46	tmo	ThumbPalm
47	pcip	PalmWrist
48	ip	IndexPalm

49	n.a.	n.a.
50	n.a.	n.a.
51	pcmp	PalmWrist
52	n.a.	n.a.
53	n.a.	n.a.
54	rdi	RingTip
55	ldi	PinkyTip
56	n.a.	n.a.
57	lmo	PinkyMiddle
58	pcmd	PalmWrist
59	ldo	PinkyTip
60	pdl	PalmFinger
61	pdr	PalmFinger
62	pdlo	PalmFinger
63	lpo	PinkyPalm
64	n.a.	n.a.
65	n.a.	n.a.

Supplementary Table 6: Description of the .tsv files

	**Name**	**Type**	**Units**	**Sampling frequency**	**Tracked point**	**Component**
Description	Channel label	Type of data	S.I. units	Sampling rate of the channel [Hz]	Label of the tracked point on the body	Representational axes in the motion system
Cometa	See column *Abbreviation* of Supplementary Table 2	EMG	[mV]	2000	n/a	n/a
OTB Sessanta quattro	See column *Abbreviation* of *Supplementary Table 1*	EMG	[mV]	2000	n/a	n/a
Vicon	See column *Abbreviation* of Supplementary Table 3	JNT ANG	[DEG]	100	See column *Description* of Supplementary Table 3	X Y Z
Cyberglove	See column *Abbreviation* of Supplementary Table 4	JNT ANG	[DEG]	100	See column *Description* of Supplementary Table 4	n/a
Tactile glove	See column *Abbreviation* of Supplementary Table 5	FORCE	[N]	100	n/a	n/a

Supplementary Table 7: Description of the .json files for each type of acquired signal. The symbols in the last three columns indicate the presence of each entry in the .json files of the respective folders (emg, motion or tactile in Figure 4.B).

Field name	**Data type**	**Description**	**emg**	**motion**	**tactile**
DeviceSerialNumber	string	The serial number of the acquired equipment that was used for data record	✓	✓	✓
ECGChannelCount	number	Number of electrocardiogram recorded channels	✓		
EMGChannelCount	number	Number of EMG channels	✓		
EMGGround	string	Physical placement of the ground electrode	✓		
EMGPlacementScheme	string	Methodology used for EMG electrodes placement	✓		
EMGReference	string	Type of acquisition	✓		
EOGChannelCount	number	Number of electrooculogram recorded channels	✓		
ElectrodeManufacturer	string	Manufacturer of the EMG electrodes	✓		
ElectrodeManufacturersModelName	string	Manufacturer's label of the EMG electrodes	✓		
HardwareFilters	string	Description of applied hardware filters	✓		
InstitutionAddress	string	The address of institution where the equipment was used	✓	✓	✓
InstitutionName	string	The name of institution where the equipment was used	✓	✓	✓
InstitutionDepartmentName	string	The name of department in the institution where the equipment was used	✓	✓	✓
Manufacturer	string	Manufacturer of the acquired system	✓	✓	✓
ManufacturesModelName	string	Manufacturer's label of the acquired hardware system	✓	✓	✓
PowerLineFrequency	number	Frequency of the power grind in Hz	✓		
MotionChannelCount	number			✓	
RecordingDuration	number	Length of the record in seconds	✓	✓	✓
RecordingType	string	Type of record	✓	✓	✓
SamplingFrequency	number	Sampling frequency of the tracking system in Hz	✓	✓	✓
SoftwareFilters	string	Description of applied software filter	✓		
SoftwareVersion	string	Acquisition's software label	✓	✓	✓
SubjectArtefactDescription	string			✓	✓
TactileChannelCount	number	Total number of taxels			✓
TaskDescription	string	Description of the task	✓	✓	✓

Taskname	string	Name of the task	✓	✓	✓
